# Carbon Ion Therapy: A Modern Review of an Emerging Technology

**DOI:** 10.3389/fonc.2020.00082

**Published:** 2020-02-04

**Authors:** Timothy D. Malouff, Anita Mahajan, Sunil Krishnan, Chris Beltran, Danushka S. Seneviratne, Daniel Michael Trifiletti

**Affiliations:** ^1^Mayo Clinic Florida, Jacksonville, FL, United States; ^2^Mayo Clinic, Rochester, MN, United States

**Keywords:** carbon, heavy ion, particle, radiation therapy, high LET radiation

## Abstract

Radiation therapy is one of the most widely used therapies for malignancies. The therapeutic use of heavy ions, such as carbon, has gained significant interest due to advantageous physical and radiobiologic properties compared to photon based therapy. By taking advantage of these unique properties, carbon ion radiotherapy may allow dose escalation to tumors while reducing radiation dose to adjacent normal tissues. There are currently 13 centers treating with carbon ion radiotherapy, with many of these centers publishing promising safety and efficacy data from the first cohorts of patients treated. To date, carbon ion radiotherapy has been studied for almost every type of malignancy, including intracranial malignancies, head and neck malignancies, primary and metastatic lung cancers, tumors of the gastrointestinal tract, prostate and genitourinary cancers, sarcomas, cutaneous malignancies, breast cancer, gynecologic malignancies, and pediatric cancers. Additionally, carbon ion radiotherapy has been studied extensively in the setting of recurrent disease. We aim to provide a comprehensive review of the studies of each of these disease sites, with a focus on the current trials using carbon ion radiotherapy.

## Introduction

The therapeutic advantages of particle radiotherapy were first recognized by Robert Wilson in the 1940s (1). Since that time, particle therapy has enjoyed a rapid growth, with centers across the world treating with protons and other heavy ions, including carbon ions. The National Institute of Radiologic Sciences (NIRS) opened the first heavy ion accelerator for clinical use in Chiba, Japan, in 1994 (2). Since that time, over 20,000 patients have been treated with carbon ion radiation therapy (CIRT) (3). Today, there are five countries and a total of 13 centers treating with CIRT (1, 4). At NIRS, 22% of patients treated have had localized prostate cancer, with other common sites including bone and soft tissue (13%) and head and neck (11%) (5).

**Figure 1:**
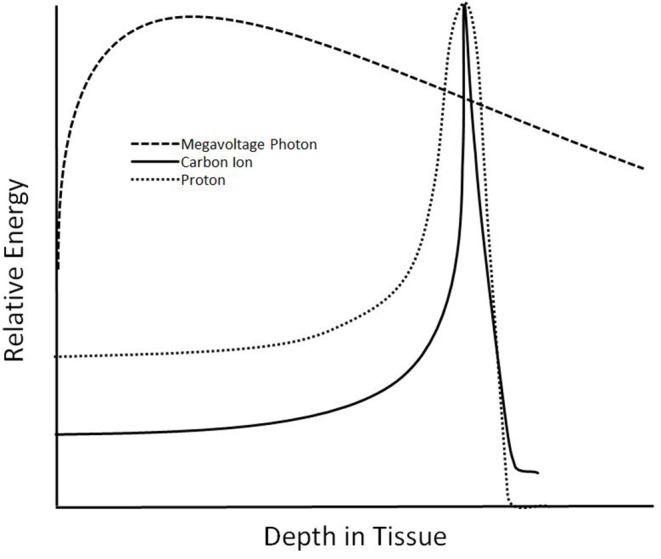
Dose deposition at depth for 6 MV photons, protons, and carbon ions.

Treatment with carbon ions provides several unique physical and radiobiologic properties (Table 1). Carbon ions exhibit a characteristic energy distribution in depth, known as the “Bragg Peak,” where low levels of energy are deposited in tissues proximal to the target, and the majority of energy is released in the target (Figure 1). Distal tissues receive little energy, although, unlike protons, there is energy deposited distally due to nuclear fragmentation (10). Additionally, a steeper lateral dose penumbra is observed at greater depths than with heavy ions, such as carbon, than with photons or protons (1, 6). Furthermore, carbon exhibit a higher linear energy transfer (LET) than photons and protons. This leads to a higher relative biological effectiveness (RBE), where damage caused by carbon ions is clustered in the DNA, overwhelming the cellular repair systems (6). With a higher LET than other methods of radiation and the characteristics of the Bragg Peak, CIRT provides a promising treatment choice for providing higher doses to targets while reducing irradiation to organs at risk (OARs).

**Table 1:** Comparison between photon, proton, and carbon-based radiotherapy.

	**Carbon ions**	**Protons**	**Photons**
Year of first treatment	1994^a^	1954	Late 1800s and early 1900s^b^
Number of sites treating (as of June 2019)	12^c^	83^d^	Routine
Bragg-Peak	Present	Present	Absent
Estimated RBE^a^	2.5–5.0	1.1	1.0
Relative LET	Highest	High	Low
Relative risk of secondary malignancy^e^^,^^f^	Low	Low	High

*RBE, relative biologic effectiveness; LET, linear energy transfer*.

a *Mohamad et al. (6)*.

b *Gianfaldoni et al. (7)*.

c *Lazar et al. (3)*.

d *Particle Therapy Co-Operative Group (4)*.

e *Eley et al. (8)*.

f *Mohamad et al. (9)*.

Centers treating with CIRT can take advantage of two types of treatment techniques to help conform the dose distribution to minimize dose to OARs. Similar to proton therapy, CIRT can be delivered either through passive scattering (using a collimator to shape the beam in the lateral direction and a range compensator to shape the beam distally) or active scanning (using a narrow “pencil beam” that avoids the use of a collimator or compensator) (11). Notably, use of active scanning, along with the physical properties of the Bragg Peak and a slower treatment time, may lead to higher uncertainty due to physiologic motion. Mitigating the dose uncertainty due to motion is an active area of research.

Due to the size and expense associated with CIRT, the majority of centers are treating with fixed-beam gantries, limiting the treatment positions available and requiring changes in patient setup prior to irradiation with multiple beams. Currently, there are two centers with rotating gantries, thus with the ability to irradiate from all angles (1). The reader is referred to the text *Carbon Ion Radiotherapy: Principles, Practices, and Treatment Planning* by Tsujii et al. for a more thorough discussion regarding the technical aspects of treatment (11).

To date, there have been multiple textbooks and review articles summarizing the current radiobiological, physics, and clinical knowledge of CIRT (1–3, 5, 6, 11–20). In this review, our aim is to provide a comprehensive and updated summary of the current clinical literature for patients treated with carbon ion radiotherapy, with an emphasis currently accruing studies. An overview of the current trials is provided in Table 2.

**Table 2:** Review of current trials evaluating carbon ion radiation therapy (CIRT).

**Trial**	**Location**	**Population**	**Arms**
**CENTRAL NERVOUS SYSTEM**			
MARCIE trial (NCT01166321)	HIT	Simpson grade 4–5 meningioma	A: 48–52 Gy photons with 18 GyE/6 fraction CI boost
PINOCCHIO trial (NCT01795300)	HIT	Skull base meningioma	A: Conventionally fractionated radiation (57.6 Gy/32 fractions) B: Hypofractionated photon (45 Gy/15 fractions) C: Proton (45 GyE/15 fractions) D: CIRT (45 GyE/15 fractions)
CLEOPATRA (NCT01165671)	HIT	Primary glioblastoma	A: 48–52 Gy photons with 18 GyE/6 fraction CI boost B: 48–52 Gy photons with 10 GyE/5 fraction proton boost
CINDERELLA (NCT01166308)	HIT	Recurrent gliomas	A: Fractionated stereotactic radiotherapy (up to 36 Gy/18 fractions) B: CIRT (dose escalating 30–48 GyE/10–16 fractions)
**SKULL BASE**			
CSP12C (NCT01182753)	HIT	Skull base chondrosarcoma	A: CIRT 60 GyE B: Proton 70 GyE
HIT-1 (NCT01182779)	HIT	Skull base chordoma	A: CIRT 63 GyE B: Proton 72 GyE
**HEAD AND NECK**			
COSMIC (NCT01154270)	HIT	Salivary gland tumors with inoperable, N+, residual disease	A: 50 Gy IMRT followed by CI boost (24 GyE/8 fractions)
Trial evaluating particle therapy with or without apatinib for H&N adenoid cystic carcinoma (NCT02942693)	SPHIC	Adenoid cystic carcinoma	A: Proton therapy (56 GyE/28 fractions) with CI boost (15 GyE/5 fractions) A: Proton therapy (56 GyE/28 fractions) with CI boost (15 GyE/5 fractions) with apatinib
ACCEPT (NCT01192087)	HIT	Adenoid cystic carcinoma	A: Combination IMRT with CI boost and erbitux
IMRT-HIT-SNT (NCT01220752)	HIT	Sinonasal tumors	A: IMRT in 2 Gy per fraction and CI boost (24 GyE/8 fractions)
**GASTROINTESTINAL**			
Trial evaluating carbon ion radiation therapy for locally recurrent nasopharyngeal carcinoma (NCT02795195)	SPHIC	Locally recurrent nasopharyngeal carcinomas	A: Dose escalation (54–63 GyE in 3 GyE daily fractions)
PROMETHEUS-01 (NCT01167374)	HIT	HCC	A: Dose escalation (40–56 GyE/4 fractions)
Phase II trial of carbon-ion radiotherapy combined with GM-CSF for the treatment of hepatocellular carcinoma (NCT02946138)	SPHIC	HCC	A: Hypofractionated carbon ion radiation (40 GyE/5 fractions) with GM-CSF
Proton and carbon ion radiotherapy for locally advanced pancreatic cancer (NCT03949933)	SPHIC	Locally advanced pancreatic cancer	A: Proton therapy to 50.4 GyE/28 fractions with a CI boost (12–18 GyE in 3 GyE per fraction)
PIOPPO (NCT03822936)	CNAO	Resectable or borderline resectable pancreatic adenocarcinoma	A: Neoadjuvant FOLFIRONX followed by 38.4 GyE/8 fractions CIRT. Resection and adjuvant gemcitabine
CIPHER (NCT03536182)	UT Southwestern (treatment in Japan)	Unresectable pancreatic cancer	A: CIRT in 12 fractions with concurrent gemcitabine and adjuvant gemcitabine and nab-paclitaxel B: IMRT in 28 fractions with concurrent gemcitabine and adjuvant gemcitabine and nab-paclitaxel
PANDORA-1 (NCT01528683)	HIT	Recurrent and inoperable rectal cancer	A: Dose escalation (36–54 GyE in 3 GyE fractions)
**GENITOURINARY**			
Carbon ions boost followed by pelvic photon radiotherapy for high risk prostate cancer (NCT02672449)	CNAO	High risk prostate cancer	A: CI boost followed by pelvic IMRT to 45 Gy
Carbon ion radiotherapy in treating patients undergoing systemic therapy for oligo-metastatic prostate cancer (NCT02935023)	SPHIC	Oligo-metastatic prostate cancer	A: CIRT to the prostate (59.2 GyE/16 fractions) with hormone therapy or chemotherapy
**SARCOMA**			
ISAC (NCT01811394)	HIT	Sarcococcygeal chordoma	A: Proton irradiation (4 GyE × 16 fractions) B: CIRT (4 GyE × 16 fractions)
SARCO (NCT02986516)	Italian Sarcoma Group	Sacral chordoma	A: Surgery with or without radiation (including CIRT) B: Definitive radiation

*CI, carbon ion; CNAO, National Center of Oncological Hadronotherapy, Italy; HCC, hepatocellular carcinoma; HIT, Heidelberg Ion Therapy Center; IMRT, intensity modulated radiation therapy; N+, node positive; SPHIC, Shanghai Proton and Heavy Ion Center; UT, University of Texas*.

## Intracranial Tumors

### Meningioma

Multiple studies have confirmed the safety and favorable toxicity profile of carbon radiotherapy for intracranial malignancies, with early use focusing on delivering a carbon ion boost following conventional photon or proton therapy (18, 21–24). A preliminary study at the Heidelberg Ion Therapy Center (HIT) evaluated 10 patients with high-risk meningioma treated with photon based radiotherapy and a carbon ion boost to a dose of 18 GyE, with local control (LC) of 72% at 7 years (25, 26).

The phase II MARCIE trial (NCT01166321) is further investigating the use of carbon ion boost in patients with residual disease following surgical resection (Simpson Grade 4–5). Enrolled patients receive 48–52 Gy of photon therapy followed by an 18 GyE carbon ion boost given in 6 fractions (27). The PINOCCHIO study is a four-arm trial investigating conventionally fractionated photon therapy, hypofractionated photon therapy, proton therapy, and CIRT for the treatment of skull base meningioma (NCT01795300).

### High Grade Glioma

High grade gliomas are typically radioresistant, with a poor prognosis despite aggressive treatment (26, 28, 29). Because of this, there has been significant interest in using CIRT as a novel treatment strategy for patients with glioma. Mizoe et al. reported on 48 patients treated with 50 Gy of photons followed by a carbon ion boost of 16.8–24.8 GyE in 8 fractions with nimustine hydrochloride. Overall survival was improved in patients receiving a higher carbon boost dose, with a median progression free survival (PFS) of 26 months. Notably, there were few grade 3 or higher acute events and no late grade 3 toxicities (24, 30). Stemming from data collected in this trial, simulated survival curves were generated for patients treated with both temozolomide and CIRT, with a potential benefit to concurrent therapy identified (31). This is currently being investigated as part of the CLEOPATRA trial.

The CLEOPATRA trial is a phase II study at HIT evaluating the use of a carbon ion boost or proton boost after concurrent photon therapy and temozolomide for primary glioblastoma. Patients are treated with 48–52 Gy of photons followed by a carbon boost of 18 GyE in 6 fractions or 10 GyE in 5 fractions in the proton group (32).

In the setting of disease recurrence, the CINDERELLA trial is a randomized, phase I/II study comparing carbon and fractionated radiation therapy for progressive or recurrent gliomas. This trial, which has recently completed accrual, treated patients with escalating dose from 30 GyE in 10 fractions to 48 GyE in 16 fractions and 36 Gy in 18 fractions in the photon group (33).

### Skull Base Chordoma/Chondrosarcoma

Skull base tumors present a challenge for treatment given their proximities to OARs. CIRT thus provides a theoretical advantage for treatment. Mizoe et al. reported on three protocols from NIRS, where a dose of 60.8 GyE in 15 fractions was given over 4 weeks. The 5-year LC was 100% without excessive toxicity (34). Further, with a median dose of 60 GyE in 20 fractions, HIT reported on 96 patients with a 70% 5-year LC. Late grade 3 optic neuropathy was seen in 4.1% of patients, and temporal lobe injury in 7.2% (35). In nine patients treated with resection and adjuvant CIRT, the 7-year overall survival (OS) was 85.7% and the 3-year and recurrence free survival (RFS) rate was 70.0% (36).

For skull base chondrosarcoma, a LC of 96.2% at 3 years and 89.8% at 4 years for patients with low and intermediate grade disease was seen following treatment to a dose of 60 GyE. One patient developed acute grade 3 mucositis with no other acute grade 3 toxicity. One patient had late grade 3 toxicity (37).

In order to compare clinical outcomes following proton and carbon ion therapy in the treatment of low grade skull base chondrosarcomas, HIT opened a randomized trial comparing 60 GyE in 20 fractions of CIRT and 70 GyE in 2 GyE per fraction of proton therapy (NCT01182753) (38). A similar trial is open comparing proton therapy (72 GyE in 2 GyE per fraction) to CIRT (63 GyE in 3 GyE per fraction) for the treatment of skull base chordoma (NCT01182779).

### Uveal Melanoma

Tsuji et al. reported on 59 patients with locally advanced or unfavorably located choroidal melanoma treated at NIRS from 2001 to 2006. Patients were treated with a single anterior field to doses between 60 GyE and 85 GyE, each given in five fractions, with a 3-year LC of 97.4%. Overall, 40% of patients developed neovascular glaucoma, mostly in the high dose group, with three requiring enucleation (5% of all patients) (39).

### Orbital Tumors

HIT evaluated 24 patients with radioresistant malignant lacrimal gland tumors treated with active raster scanning technique. The median local control was 24 months, with no grade 4 or higher toxicity (40). NIRS reported on 33 patients with lacrimal gland tumors with extraorbital extension treated to either 57.6 GyE or 64 GyE in 16 fractions. Although there was an 86% ipsilateral eye preservation rate, 36.4% of patients developed grade 4 optic nerve disorders (41).

## Head and Neck Tumors

### Adenoid Cystic Carcinoma

Early results from HIT reported a 3-year LC rate of 62% for the 21 patients with unfavorable adenoid cystic carcinomas treated with combination photon and CIRT. No grade 3 or 4 toxicities were observed (17). Sixteen patients with locally advanced adenoid cystic carcinoma were enrolled in a phase I/II trial photon and CIRT boost to a dose of 72 GyE. Three-year LC was 64.6% with no patient developing grade 3 or higher complications (42).

A subanalysis of J-CROS 1402 HN patients found a 3-year LC and OS of 81 and 94% with a median dose of 64 GyE in 16 fractions. Two patients experienced late grade 3 toxicity of dysphagia and brain abscess (43). A prospective study analyzing 35 patients at Gunma University Heavy Ion Medical Center for non-squamous cell carcinomas of the head and neck showed promising results with patients receiving 64 GyE and 57.6 GyE in 16 fractions, with 3-year LC and OS rates of 93 and 88%, respectively (44).

The COSMIC trial is a phase II trial evaluating combined IMRT to a dose of 50 Gy followed by carbon ion boost to 24 GyE over 8 fractions for patents with salivary gland tumors with inoperable, node positive, or residual disease (NCT01154270). Apatinib is also being investigated with proton therapy followed by a carbon ion boost for adenoid cystic carcinomas (NCT02942693). The ACCEPT study is a phase II trial of combination IMRT followed by carbon ion boost with cetuximab for adenoid cystic carcinoma, and is currently open at HIT (NCT01192087).

### Parotid Gland Tumors

Patients treated for locally advanced parotid gland tumors with CIRT at NIRS showed a 5-year local control of 74.5% and overall survival of 70.1%. Of the 30 patients without facial nerve deficits prior to radiation, 25 continued to have no evidence of radiation induced facial nerve damage (45).

### Nasopharyngeal Cancer

In particular, nasopharyngeal carcinomas are among the most accepted indications and may benefit the most from particle therapy, as they often abut critical OARs like the brainstem, optic apparatus, and temporal lobes. HIT retrospectively analyzed 26 patients with high risk nasopharyngeal cancer treated with IMRT and carbon ion boost for a cumulative dose of 74 Gy RBE. With a median follow up of 40 months, 60% had a complete response, with 20% demonstrating partial response and 12% stable disease. The 2-year OS, LC, and distant progression-free survival (PFS) rates were 100, 95, and 93%, respectively. Acute grade 3 toxicity was seen in 20% of patients, with 16% developing late grade 3 toxicity. There were no grade 4 or 5 toxicities (46).

Akbaba et al. described 59 patients with adenoid cystic carcinoma of the nasopharynx treated at the Heidelberg Ion-Beam Therapy Centers with combination photon and carbon ion boost radiation therapy. Patients were treated to a dose of 50–56 Gy IMRT followed by 18–24 GyE boost with carbon. The 2-year OS and LC were 87 and 83%, with 12% acute and 8% late grade 3 toxicity (47).

In the setting of recurrent disease, CIRT to a dose of 50–66 GyE with varying fractionation schedules (between 2 and 3 GyE per fraction) delivered by raster scanning has shown promising results, with a 1-year PFS and OS were both 98%. Grade 3 and 4 late toxicities included mucosal necrosis (9%), xerostomia (1%), and temporal lobe necrosis (1%) (48). Additionally, the Shanghai Proton and Heavy Ion Center (SPHIC) is evaluating the maximum tolerated dose of retreatment using raster scanning CIRT with concurrent cisplatin (49).

### Sinonasal Cancer

Koto et al. investigated 22 patients with sinonasal adenocarcinoma treated with CIRT either as definitive therapy or following surgery or chemotherapy, with 14 patients receiving 57.6 GyE in 16 fractions and 8 receiving 64.0 GyE in 16 fractions. With a median follow up of 43 months, the 3-year LC and OS were 76.9 and 59.1%, respectively. Notably, five patients experienced lateral vision loss. Symptomatic brain necrosis and mucosal ulceration were observed in one patient each, respectively (50).

HIT is currently investigating IMRT followed by a 24 GyE in 8 fraction boost to inoperable or residual disease (NCT01220752). A dose escalation study of CIRT for recurrent nasopharyngeal carcinoma is currently open at the SPHIC, with doses from 55 to 65 GyE at 2.5 GyE per day (NCT02795195).

### Otic Cancer

Primary otic tumors are extremely rare with a poor prognosis, with surgery the mainstay of treatment. JCROS evaluated 31 patients treated for external auditory canal or middle ear carcinomas, with a median dose of 64 GyE in 15 fractions. Three-year OS was 58.7% with similar LC rates. Grade 3 dermatitis was seen in 9.7% of patients, with central nervous system (CNS) necrosis in 6.5%. There were no grade 4 or 5 toxicities (51). Further, NIRS reported on 13 patients with a 3-year LC of 56.4% and OS of 41.6%. Two patients had severe temporal bone necrosis, and four patients developed grade 1–2 localized brain necrosis (52).

### Oral Cancer

In a large retrospective series, Ikawa et al. analyzed 76 patients with non-squamous oral cavity cancers treated across four institutions in Japan from 2004 and 2014. Forty-six patients had salivary gland carcinoma and 27 had mucosal melanoma. With a median follow up of 31.1 months, the 3-year LC, PFS, and OS were 86.6, 63.1, and 78.4%, respectively. Thirteen patients had late grade 3 or higher toxicity, with 9 patients having grade 3 osteoradionecrosis. There were no grade 5 toxicities. The authors conclude that carbon ion radiotherapy is effective with “acceptable” toxicity for oral cavity cancers (53).

### Recurrent Disease

Re-irradiation with CIRT appears to be a reasonable treatment option in patients who developed recurrent disease after primary CIRT. SPHIC reported on 19 patients with recurrent or radiation induced sarcoma of the head and neck treated with CIRT to a median dose of 60 GyE. The 12 month survival was 86.5%, with two grade 4 toxicities (acute hemorrhage from the sphenopalatine artery). There were no grade 5 toxicities (54).

NIRS investigated 48 patients with locoregional failure previously treated with a mean dose of 57.6 GyE in 12 fractions using CIRT. With a median dose of 54 GyE, 10.4% of patients developed grade 3 acute toxicity and 37.5% of patients developed grade 3 or higher late toxicity. There was one grade 5 toxicity. The 2-year LC and OS rates were 40.5 and 59.6% (55). Further, SPHIC reported on 19 patients with recurrent or radiation induced disease treated to 60 GyE. No grade 5 toxicities were seen (54).

## Lung Tumors

### Non-small Cell Lung Cancer (NSCLC)

Dosimetric studies have shown lower OAR doses and a more homogenous target dose for NSCLC with CIRT compared to photon, potentially allowing for hypofractionation without increasing toxicity (56). In localized disease, Miyamoto et al. described 47 patients in a dose escalation trial with doses from 59.4 to 95.4 GyE. Grade 3 radiation pneumonitis occurred in three patients, although this was not dose limiting as defined by the protocol (57). Hypofractionation was then attempted to a dose of 72 GyE in 9 fractions, with a 94.7% LC rate and no grade 4–5 toxicity (58). In a second hypofractionation study, patients with stage IA were treated to 42.8 GyE and stage IB to 60.0 GyE in four fractions. The local control was 98% for T1 and 80% for T2 tumors. No grade 4 or 5 lung toxicities were seen (59). Single fraction CIRT appears to be feasible for early stage NSCLC with LC of 95% at 5 years with doses above 48 GyE (60).

For locally advanced NSCLC, hypofractionated dose escalation above 76 GyE resulted in unacceptable toxicity, and the recommended dose was 72 GyE in 16 fractions (61). Further, a phase I study from the Gunma University Heavy Ion Medical Center treated unresectable stage III NSCLC with a hypofractionated region of 54 GyE in 4 GyE daily fractions. Of the 6 patients that were treated, the overall response rate was 100% with no dose limiting toxicity (62). One study using 72 GyE in 16 fractions had seven (out of 141 patients) cases of grade 4 toxicity, including mediastinal hemorrhage, radiation pneumonitis, or bronchial fistulas (63).

A diagnosis of interstitial lung disease presents a challenge to radiation oncologists, as radiation can cause an exacerbation of the underlying lung disease. Notably, CIRT was found to be low risk in patients with lung disease, with only two of 29 patients (6.9%) experiencing an exacerbation (64).

## Gastrointestinal Tumors

### Esophageal Cancer

CIRT has been used successfully in the treatment of squamous cell carcinoma of the esophagus. In a phase I/II trial, 31 patients with resectable disease received between 28.8 and 36.8 GyE in 8 fractions given 4 fractions per week. A 38.7% pathologic complete response rate and 41.9% clinical partial response rate were seen (65).

### Hepatocellular Carcinoma (HCC)

In Japan, two protocols (9603 and 0004) attempted hypofractionated treatment for HCC. 52.8 GyE in 4 fractions was the recommended dose, with a 5-year LC of 90% (66). Recent studies have attempted further dose-escalation, with 48.0–60 GyE given in 4 treatments. All doses had favorable LC and survival rates. Notably, 5.7% had grade 3–4 toxicity with 1.7% developing radiation induced liver disease (67). Given the safety of the four fraction regimens, these were applied to 21 patients with HCC lesions >3 cm, with similar LC and toxicity rates (68). Further, treatment with 15 fractions in patients with cirrhosis did not increase the Child-Pugh score by more than 2 points (69). Patients treated with 52.8 GyE in 4 fractions had no difference in OS or LC based on if the tumor was within 2 cm of the porta hepatis (70). There was also no difference in toxicity. Given this, the hypofractionated course is felt to be safe for treating in close proximity to the portal system.

In a propensity score matched review, 477 patients were treated with either CIRT or transarterial chemoembolization for treatment naive, single tumor hepatocelluar carcinoma. Doses of 52.8–60 Gy in 4 fractions were used, with 60.0 Gy in 12 fractions close to the GI tract. Treatment with CIRT showed improved OS (88% vs. 58%), LC (80% vs. 26%), and PFS (51% vs. 15%) compared to TACE for single tumor HCC (71).

The PROMETHEUS-01 trial (NCT01167374) is a phase I study evaluating CIRT in advanced HCC without evidence of extrahepatic disease. Patients will be treated at increasing doses from 40 GyE in 4 fractions to 56 GyE in 4 fractions (72). A current phase II study at SPHIC is investigating the use of hypofractionated CIRT to a dose of 40 GyE in 5 fractions with GM-CSF in the treatment of HCC (NCT02946138).

### Liver Metastases

Makishima et al. found a 3-year LC rate of 82% for single fraction treatment for colorectal cancer liver metastasis at doses above 53 GyE, compared to 28% at lower doses. In contrast to the above study, there were two cases of grade 3 liver toxicity at 53 GyE, with both cases occurring near the hepatic portal region. The authors conclude that single fraction therapy is safe up to 58 GyE if the central hepatic portal region can be avoided (73).

### Cholangiocarcinoma

The Japan Carbon Ion Radiation Oncology Study Group (J-CROS) investigated the role of CIRT for 56 patients with intrahepatic (27 patients) and perihilar (29 patients) cholangiocarcinoma. No patients underwent resection. Most patients were treated to a dose of 76 GyE in 20 fractions, with a median survival of 23.8 months for intrahepatic cholangiocarcinoma and 12.6 months in perihilar disease. Notably, there was one case of grade 5 liver injury and one grade 3 bile duct stenosis (74).

### Pancreatic Cancer

Shinoto et al. evaluated the safety and efficacy of short course, neoadjuvant irradiation with CIRT for potentially resectable pancreatic cancer. The dose given was escalated from 30 to 36.8 GyE in 8 fractions, with surgery performed up to 4 weeks after completion of radiation. Sixty-five percentage of the 28 patients developed distant disease, with no patients experiencing local recurrence. One patient developing acute grade 3 liver toxicity and one patient developing late grade 4 portal vein stenosis (75).

The J-CROS Study 1403 retrospectively analyzed 72 patients from three institutions with locally advanced pancreatic adenocarcinoma and treated with CIRT to a dose of 52.8 GyE or 55.2 GyE in 12 fractions. The median OS was 21.5 months. The 2-year local recurrence rate was 24%. Twenty-six percentage of patients experienced grade 3 or four hematologic toxicities with 3% grade 3 anorexia (76).

The PHOENIX-1 trial was a phase I study at HIT evaluating the CIRT using raster scanning in combination with weekly gemcitabine and adjuvant gemcitabine in the treatment of locally advanced pancreatic cancer, although the trial is currently on hold due to “administrative barriers.” The dose escalation study aimed to determine the safety of CIRT ranging from 42 GyE in 14 fractions to 54 GyE in 18 fractions (77).

A phase I trial at SPHIC is investigating the use of 50.4 GyE in 28 fractions followed by a carbon ion boost of doses from 12 to 18 GyE in 3 GyE per fraction (NCT03949933). The PIOPPO study, a phase II study of neoadjuvant FOLFIRINOX followed by 38.4 GyE in 8 fractions over 2 weeks followed by resection and adjuvant gemcitabine for patients with resectable or borderline resectable pancreatic cancer, is currently accruing (NCT03822936).

In Japan, 64 patients with unresectable pancreatic cancer were treated with 55.2 GyE in 12 fractions, with a median survival of 25.1 months and 2-year LC of 82%. Four patients had acute grade 3 toxicity (78).

The CIPHER study, sponsored by the University of Texas Southwestern Medical Center, is a phase III trial comparing IMRT with CIRT for unresectable pancreatic cancer. Both arms receive concurrent gemcitabine followed by adjuvant gemcitabine and nab-paclitaxel. Patients randomized to the CIRT are flown to centers with CIRT for treatment (NCT03536182).

### Rectal Cancer

Patients with a rectal cancer recurrence following primary curative intent therapy were treated with a dose of 73.6 Gy RBE in 16 fractions as part of the GUNMA 0801 prospective study (79). The 3-year OS, LC, and PFS for the 28 patients were 92, 86, and 31%, respectively. Similarly, the JCROS experience found that patients treated to 70.4 GyE or 73.6 GyE in 16 fractions for recurrent rectal cancer had 5-year OS rates of 51% and LC of 88%. Three patients had grade 3 toxicity with no grade 4 or 5 toxicities (80).

The PANODRA-01 phase I-II study at HIT (NCT01528683) is a dose escalation trial in the setting of recurrent and inoperable rectal cancer. Increasing doses from 36 to 54 GyE given in 3 GyE fractions will be given. Patients previously received 20–60 Gy of photon radiotherapy as part of their primary treatment (81).

## Genitourinary Tumors

### Prostate Cancer

J-CROS 1501 PR was the first multi-institutional observation study (NIRS, Gunma, and the Ion Beam Therapy Center in Saga, Japan) analyzing outcomes of prostate cancer patients treated with CIRT. Fifty-six percentage were high risk, 31% intermediate risk, and 12% were low risk. The 5-year biochemical relapse free survival (bRFS) rates were 99, 100, and 100%, respectively (82). SPHIC investigated 64 patients with localized prostate cancer treated to a dose between 59.2 and 66 GyE in 16–24 fractions without nodal irradiation. Urinary irritation declined temporarily, with quality of life scores returning to baseline at 1-year. The rates of acute grade 1 and 2 genitourinary (GU) toxicity were 20.3 and 10.9%, respectively. Late grade 1 and grade 2 GU toxicity were 3.1 and 1.6%, respectively. Notably, there was a 0% rate of late GI toxicity (83).

Similarly, patients with high risk or very high risk prostate cancer who received CIRT with long term androgen deprivation therapy had a 10-year prostate cancer specific mortality rate of 4.3%. The 10-year incidence of grade 2 GU toxicity was 11.7%, with grade 3 GU toxicity occurring in 0.5% of patients (84).

In a recent paper analyzing patients from NIRS and the Osaka registry, Mohamad et al. determined the risk of secondary malignancy was lower for patients treated with CIRT compared to conventional photon therapy (HR 0.81) or surgery (HR 0.80) for localized prostate cancer (9). A phase II trial evaluating 45 Gy IMRT to the pelvic lymph nodes, prostate, and seminal vesicles followed by a carbon ion boost is currently opened in 2016 in Italy (NCT02672449). Carbon ion therapy is currently being investigated in a phase II trial in the setting of oligometastatic prostate cancer (NCT02935023).

### Renal Cell Carcinoma

CIRT has been used with good efficacy and safety for the treatment of primary renal cell carcinoma. The NIRS experience reported on 19 patients treated with 12 or 16 fractions CIRT, with 5-year cancer specific survival (100%) and LC rates (94.1%) (85). A non-randomized phase I/II study determined that 72 GyE was well-tolerated, with no dose limiting toxicity. Cancer specific survival rates and LC were 100% with a median follow up of 43.1 months (86).

## Sarcoma

### Osteosarcoma

Osteosarcomas of the trunk are traditionally challenging to treat, as surgical resection can lead to significant morbidity. Given this, Matsunobu et al. retrospectively analyzed 78 patients with medically inoperable osteosarcoma of the trunk treated with CIRT to a median dose of 70.4 GyE in 16 fractions over 4 weeks. The 5-year LC and OS were 62 and 33%, respectively. Three patients required skin grafts, although there were no other severe late toxicities. Eight out of nine patients (89%) who were disease free for 5 years were able to ambulate with or without a walker (87).

### Extremity Sarcoma

CIRT has also been studied in localized primary sarcomas of the extremities. A phase I/II study enrolled 17 patients with either primary or recurrent disease and were treated to 52.8, 64, or 70.4 GyE in 13–16 fractions. LC and OS at 5 years was 76 and 56%, respectively. One patient had a femoral fracture, with no other grade 3 or higher late reactions (88). In a dose escalation study, Kamada et al. found a high rate of grade 3 acute skin reactions (7/17 patients) with 73.6 GyE, and dose escalation was stopped at that time (89).

### Sacral Chordoma

A total of 95 patients with medically inoperable sacral chordoma were treated in Japan between 1996 and 2007. The carbon ion dose ranged from 52.8 to 73.6 GyE, with a median dose of 70.4 GyE over 16 fractions. The OS at 5 years was 86% with a LC rate of 88%. Ninety-one percentage of patients were able to ambulate without aide. Of note, two patients required skin grafts and 15 had severe sciatic nerve complications requiring indefinite medications (90).

A phase II trial at HIT is currently investigating the use of hypofractionated irradiation with protons or CIRT after R2 resection for patients with sacrococcygeal chordoma (NCT01811394). The SACRO study is a phase III randomized trial investigating the use of surgery or definitive radiation therapy, including carbon ion radiotherapy, in the treatment of sacral chordoma (NCT02986516).

## Cutaneous Tumors

### Skin Cancer

In a Chinese series, 45 patients with squamous cell carcinoma (16 patients), basal cell carcinoma (12 patients), melanoma (8 patients), Bowen's disease (8 patients), or Paget's disease (2 patients) were treated with various dosages. Non-melanoma skin cancers were treated with 60–70 GyE, melanoma 61–75 GyE, Bowen's disease 60 GyE, and Paget's 42.5 GyE over 6–11 fractions. CIRT had favorable local control rates at 1 year, ranging from 80 to 90% for all histologies (91).

### Keloids

CIRT has been successfully used as adjuvant therapy in the treatment of keloids. In a case series from China, 16 patients with keloids were treated postoperatively with 16 GyE in 8 fractions. A 95% success rate was achieved with a mean follow up of 29.7 months. There was no grade 3 or higher toxicities (92).

## Breast Cancer

There is currently a lack of clinical data investigating the use of CIRT in breast cancer. To date, there are no large clinical trials using CIBT. In a phase I dose escalation study, Karasawa et al. analyzed 7 patients who underwent CIBT who then underwent tumor excision for pathologic evaluation. Three patients received 48 GyE, three received 52.8 GyE, and one received 60.0 GyE. All patients were treated in four fractions and were treated supine with cast and thermoplastic immobilization. Four patients had acute grade 1 skin toxicity, with no other reported acute toxicity. At 3 months, most patients experienced some pathologic response to treatment, although the authors concluded that 3 months was not sufficient to fully evaluate treatment response (93).

Dosimetrically, there is unlikely a significant difference in dose distribution between passive and active scanning CIRT, although there may be a slight advantage to passive scanning in some patients with unfavorable anatomy (94).

## Gynecologic Cancer

### Cervical Cancer

In a systematic review of eight clinical studies from NIRS, Wang et al. concluded that carbon ion radiotherapy is safe and effective in the treatment for gynecologic cancers. The authors found lower rates of local recurrence at doses higher than 70 GyE for cervical cancer (95).

A pooled analysis of protocols 9403 and 9702 evaluated 44 patients with stage IIIB and IVA disease between 1995 and 2000 receiving CIRT for locally advanced cervical carcinoma. Patients received 16 fractions to the whole pelvis followed by a boost of 8 fractions to a dose up to 72.0 GyE. The most severe GI toxicity occurred at doses above 60 GyE, and the authors concluded that the dose to the intestines should be limited to 60 GyE (96). In the NIRS protocol 9902, no patient treated with 72.0 GyE failed locally (97).

The NIRS protocol 0508 is a phase I/II study of prophylactic extended field (involving pelvic nodes, para-aortic nodes, ovaries, and uterus) CIRT for locally advanced squamous cell carcinoma of the cervix. Twenty-one of the 26 patients had acute grade 1 or 2 toxicity with no late grade 3 or higher toxicities reported (98). Given this, prophylactic extended field radiation was considered feasible and safe.

Protocol 1001 is a phase I/II study evaluating concurrent chemoradiation for locally advanced cervical carcinoma. Patients received weekly cisplatin at 40 mg/m^2^ and a CIRT to a dose of 74.4 GyE. Treatment was well-tolerated, with two patients developing grade 3–4 GI toxicities. In patients treated with the recommended dose of 74.4 Gy, the 2-year LC, PFS, and OS were 71, 56, and 88%, respectively (99).

CIRT has also been used in combination with a brachytherapy boost for the treatment of locally advanced cervical cancer, with no dose limiting toxicities. All patients received 36 GyE in 12 fractions to the whole pelvis followed by a local boost of 19.2 GyE in 4 fractions (100).

### Endometrial Cancer

In a pooled analysis of protocols 9704 and 9404 from NIRS, the authors found that the 5-year LC and OS were 86 and 68%, respectively. Of note, inclusion criteria included inoperable and previously untreated stage I–III endometrial carcinoma without para-aortic nodal metastasis. Radiation was given to the whole pelvis with CIRT to a dose of 36.0 GyE in 12 fractions followed by a CIRT boost to a total dose of 62.4–74.4 GyE in 20 fractions with no brachytherapy boost. Notably, the GI tract dose was limited to no more than 60 GyE. No patients experienced grade 3 or higher acute or late toxicity (101).

## Pediatric Cancer

Out of a group of 394 patients treated with CIRT in Germany between 1997 and 2007 for skull base tumors, 17 patients were under the age of 21. The primary tumor was treated in 14 patients, and three patients had recurrent tumors. Patients were treated to a dose of 60 GyE using the raster scan technique. With a median follow up of 49 months, there were no severe side effects. Only one patient experienced tumor progression from a chordoma after 5 years (21, 102).

NIRS retrospectively reviewed 26 patients from 11 to 20 years old with inoperable osteosarcoma of the trunk. A median of 70.4 GyE in 16 fractions was delivered. A promising LC was also reported, at 62.9% at 5 years, with four patients developing grade 3 or higher late toxicities. Only one patient was unable to ambulate after treatment (103).

CIRT may provide a particularly important benefit in the pediatric population. As noted in the setting of prostate cancer, CIRT appears to have a lower rate of second malignancies compared to photons (9). Furthermore, the measured dose ambient dose equivalent for passive scatter carbon beams is lower than passive scattering beams for protons (104). This property, in combination with a lower neutron dose in active scanning beams compared with IMRT or passive scattering CIRT, may contribute to a lower secondary malignancy rate with CIRT (104–106).

## Discussion

CIRT represents a promising new treatment technique, with early data suggesting that it is both safe and effective for a variety of tumors. Caution should be taken in interpreting the data; however, as there is a high degree of heterogeneity with treatments in the trials, especially compared to photon therapy. For instance, in glioblastoma, the standard of care is typically radiation therapy followed by temozolomide as per the Stupp trial, with a median survival of 14.6 months (28). There is no direct comparison for CIRT, where one trial used CIRT as a boost following 50 Gy with photons with concurrent nimustine, and the second trial retrospectively reviewed patients receiving CIRT alone, with a median survival of 18 months (24, 30, 31). This makes direct comparison difficult, although more recent trials are attempting to address these deficiencies.

Compounding this issue, many of the dose regimens are hypofractionated or unconventional, leading to a difficult direct comparison. As many of the treatment centers use only fixed-beam gantries with long treatment times, using hypofractionated regimens helps overcome logistical challenges present to delivery of radiation. An example of this is in esophageal cancer, with the Japanese treating to a dose of 52.8–55.2 GyE in 12 fractions, compared to 41.4 Gy in 23 fractions as part of the CROSS trial (76, 107). This pattern of hypofractionation is seen across disease sites, and makes direct comparison of fractionation schemes challenging.

One of the major concerns regarding CIRT, and heavy ion radiotherapy in general, is dose uncertainty. With the Bragg-Peak and sharp lateral penumbra, there is a higher susceptibility to intrafraction motion compared to photon-based therapy, where the effect of motion can be mitigated due to the “dose bath.” Further, there is higher likelihood that the Bragg-Peak be in normal tissues with intrafraction motion (108). Additionally, CIRT exhibits a fragmentation tail, where nuclear fragments contribute to dose distal to the target. This creates uncertainty to tissues distal to the target to a greater degree than proton therapy, which will be an important dosimetric principle to consider.

The primary goal of CIRT is to increase the therapeutic index and reduce normal tissue toxicity while dose escalating to the tumor. The exact radiobiologic effects on normal tissues remain unclear. Additionally, there are not well-established dose constraints for normal tissues, leading to uncertainty regarding the toxicity rates at a given dose. Further preclinical study is needed to establish and standardize normal tissue dose constraints prior to widespread use.

Uncertainty remains with the precise calculation of dose from RBE. Currently, there are three models for calculating RBE, with the local effect model being used in Europe, and the remaining two models (Microdosimetric Kinetic Model and mixed-beam model) used in Japan (109). Given this, there are no current standard doses for CIRT, as is the case of the CIPHER trial, where there are two fractionation regimens as part of the study based on if the patient is treated in Japan or Europe. A consensus on the definition and calculation of RBE for CIRT is necessary prior to more widespread adoption.

Another potential benefit of CIRT may be in combination with immunotherapy. High LET radiation has been shown to have an increased immunogenicity of radiation-induced cell death compared to photon radiation through a variety of mechanisms, thus leading to a hypothesized advantage in the setting of combined immunotherapy (110). High LET radiation, such as CIRT, has been shown to induce immune cell death at lower doses compared to photon irradiation (111). In addition, higher doses per fraction have been shown to increase the immune response following radiation, and most patients treated with CIRT are treated with a hypofractionated regimen (110, 112). The reader is referred to the review articles by Helm et al. and Durante et al. for a more detailed discussion of the exact mechanisms of heavy ion therapy and combination immunotherapy (110, 113). Although, there is currently a lack of clinical data regarding CIRT in combination with immunotherapies, this will likely be an important frontier, as more patients are treated with immunotherapy. Further pre-clinical and clinical trials will be necessary to elicit the potential benefit of CIRT in combination with immunotherapy.

Despite the promising early results, the cost of developing and maintaining a heavy-ion center has been prohibitive for adoption in United States. For instance, Pompos et al. estimates that the cost of developing a center with capacity of 1,000 patients per year is roughly twice as expensive as a proton center of the same size (108). The estimated cost for multiple room centers treating with multiple ions is ~$200 million (20). Much of this cost comes from the complexity of the system, such as the need for a synchrotron and additional shielding (108). Additionally, the cost-effectiveness of this expensive treatment remains unknown, especially in the United States current payment model. CIRT has been found to be a highly cost-effective option in Germany, specifically for the treatment of skull base chordoma (114, 115). Although performing a cost-effectiveness analysis is not currently possible using US data, the data from Germany can be extrapolated, and CIRT will likely be a cost-effective treatment option for many disease sites in the US.

Currently, there are not centers in the United States currently treating with CIRT, although interest in CIRT is increasing. Internationally, there are currently five centers planned or under construction (China, France, Japan, South Korea, and Taiwan) (4). In the US, UT Southwestern has recently opened the CIPHER trial, with one of the randomizations receiving CIRT, although patients are transported overseas for treatment. Furthermore, although not extensively studied, there is likely a high medical need for CIRT in the United States, similar to in Korea or Iran (116, 117). Given the promising early clinical data as well as the advantages of heavy ion therapy, our institution recently announced the intent to construct a heavy ion facility in Jacksonville, Florida. By opening the heavy ion facility, we hope to further research the potential benefits of CIRT.

## Conclusions

Taking advantage of the unique radiobiological and physical properties, CIRT is a promising treatment technique in the treatment of a variety of malignancies. Extensive further prospective trials are needed to define the role of carbon ion therapy in clinical practice.

## Author Contributions

TM and DT: conception and design. TM, DT, and AM: manuscript preparation. TM, DT, AM, SK, CB, and DS: editing and review.

### Conflict of Interest

The authors declare that the research was conducted in the absence of any commercial or financial relationships that could be construed as a potential conflict of interest.
